# Lodged Thrombus: A Pitfall for Diagnosing Carotid Web Using Contrast-Angiography

**DOI:** 10.7759/cureus.72237

**Published:** 2024-10-23

**Authors:** Yoshitaka Yamaguchi, Kei Miyata, Fumiki Tomeoka, Minoru Ajiki, Tatsuro Takada

**Affiliations:** 1 Department of Cerebrovascular Medicine, Teine Keijinkai Hospital, Sapporo, JPN; 2 Department of Neurosurgery, Teine Keijinkai Hospital, Sapporo, JPN

**Keywords:** carotid ultrasonography, carotid web, digital subtraction angiography, embolic stroke of undetermined source, three-dimensional computed tomography angiography

## Abstract

Carotid web, a form of fibromuscular dysplasia, involves a thin, membrane-like tissue in the carotid bulb that can cause thrombus formation and is linked to cryptogenic ischemic stroke. Diagnosis typically relies on detecting a shelf-like filling defect in digital subtraction angiography or 3D-CT angiography. We report a case of the symptomatic carotid web that could not be diagnosed using DSA or 3D-CT angiography due to a lodged thrombus but was successfully identified through carotid ultrasonography. A 44-year-old woman with a history of subarachnoid hemorrhage developed left hemiparesis and was diagnosed with acute cerebral infarction due to right middle cerebral artery occlusion. Mechanical thrombectomy achieved partial reperfusion. DSA and postoperative 3D-CT angiography showed only mild stenosis at the posterior wall of the carotid bulb. However, carotid ultrasonography revealed a membrane-like structure with a lodged thrombus, leading to a diagnosis of a carotid web. Carotid artery stenting was performed to prevent further cerebral embolism, and the postoperative course was uneventful. While digital subtraction angiography and 3D-CT angiography are regarded as the gold standards for diagnosing carotid web, they may only reveal a slight, insignificant filling defect, potentially leading to a missed diagnosis when a large thrombus is present. Carotid ultrasonography is particularly useful in such cases. As revascularization procedures with CEA or CAS are highly effective in lowering the risk of recurrent ischemic stroke in symptomatic CaW, it is crucial to avoid missed diagnoses of symptomatic CaW in ESUS cases, particularly in patients without typical vascular risk factors, through multimodal imaging, including carotid ultrasonography, and to consider revascularization to prevent further stroke recurrence.

## Introduction

Carotid web (CaW), a variant of fibromuscular dysplasia, is characterized by a thin, membrane-like tissue extending into the lumen from the posterior wall of the carotid bulb [1]. It can lead to thrombus formation due to stagnant blood flow and is increasingly recognized as a potential embolic source in cryptogenic stroke [2,3]. However, awareness of CaW remains limited. The diagnosis typically depends on morphological assessment via radiographic imaging, but it can be challenging and is often missed by routine neuroimaging techniques. We present a unique case of symptomatic CaW that was missed using digital subtraction angiography (DSA) and 3D-CT angiography, the gold standard methods for diagnosing CaW, due to a lodged thrombus, but successfully identified through carotid ultrasonography.

## Case presentation

**Figure 1:**
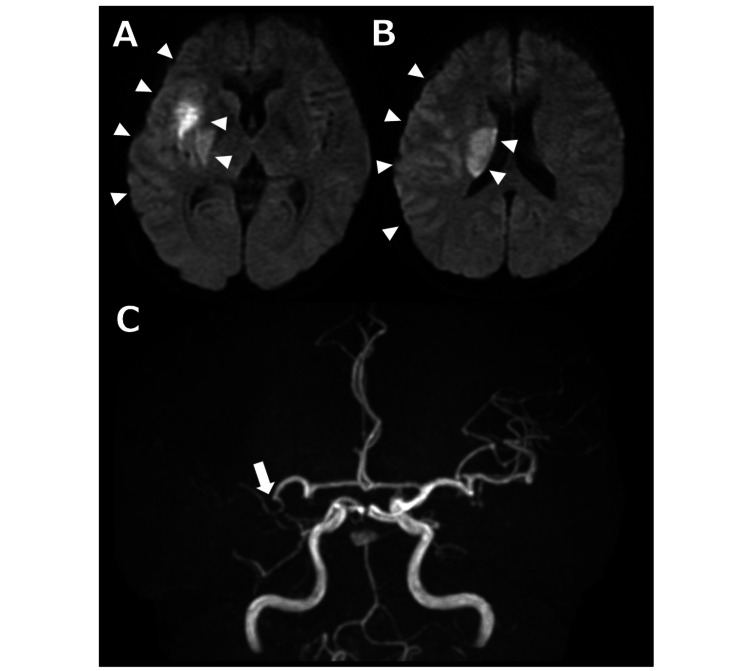
Brain MRI and magnetic resonance angiography on admission. Brain MRI (A, B) reveals high signal intensities on diffusion-weighted imaging in the territory of the right middle cerebral artery (arrowheads). Magnetic resonance angiography (C) demonstrates occlusion of the right middle cerebral artery at the distal M1 (horizontal) segment (arrow).

A 44-year-old woman was admitted to our hospital due to the sudden onset of left hemiparesis. Her medical history included a subarachnoid hemorrhage caused by a ruptured right internal carotid-posterior communicating artery aneurysm, which had been treated with surgical clipping one year prior at another hospital. Brain MRI revealed acute cerebral infarctions in the territory of the right middle cerebral artery (Figures 1A, 1B), with occlusion of the distal M1 (horizontal) segment (Figure 1C).

**Figure 2:**
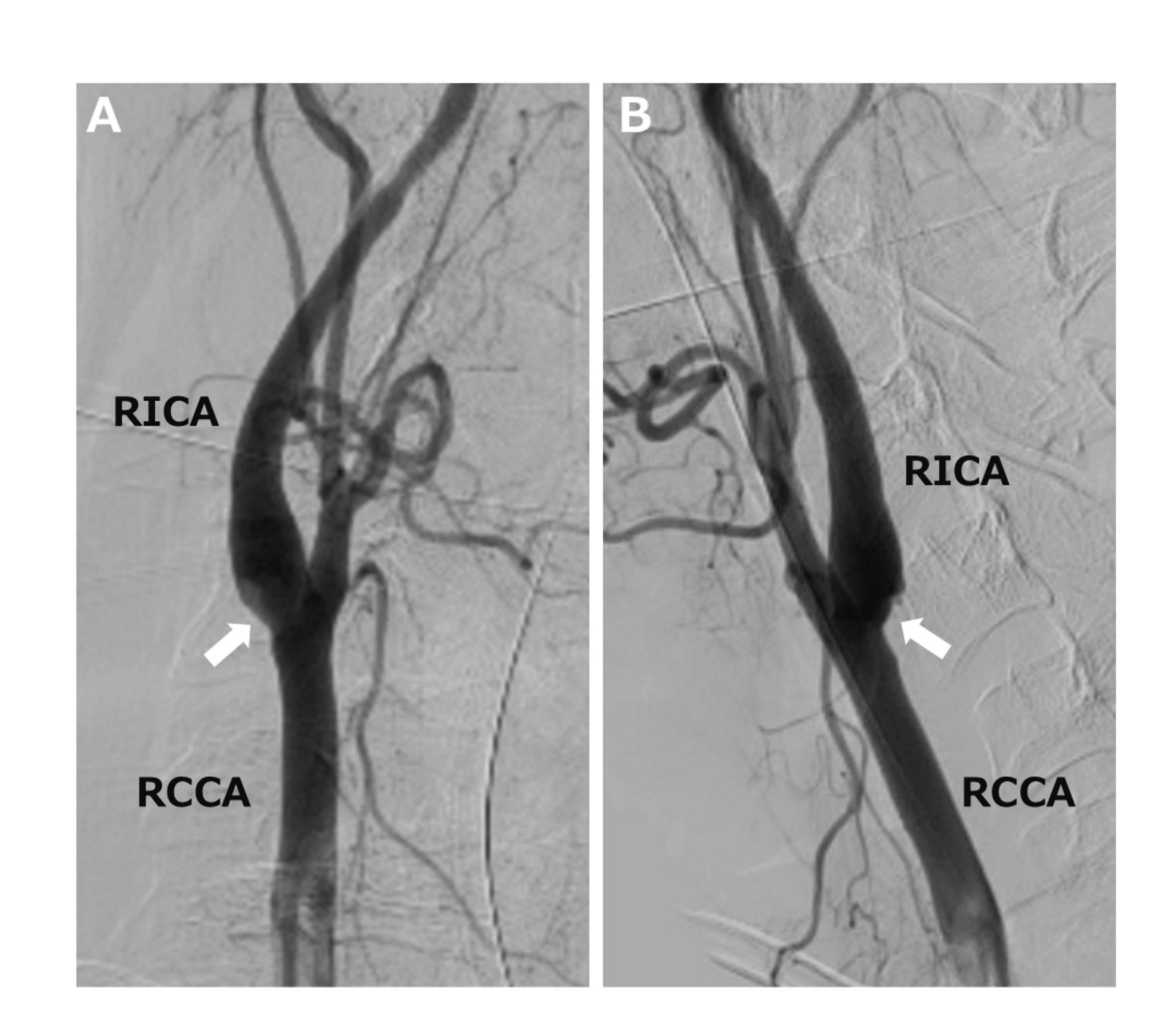
Digital subtraction angiography is performed during thrombectomy. Digital subtraction angiography (A: anterolateral view, B: lateral view) shows mild vessel wall irregularity at the posterior wall of the right carotid bulb (arrow). RCCA, right common carotid artery; RICA, right internal carotid artery

Mechanical thrombectomy achieved partial reperfusion. Post-thrombectomy right common carotid arteriography showed mild vessel wall irregularity at the posterior wall of the bifurcation of the right common carotid artery (RCCA) (Figures 2A, 2B).

**Figure 3:**
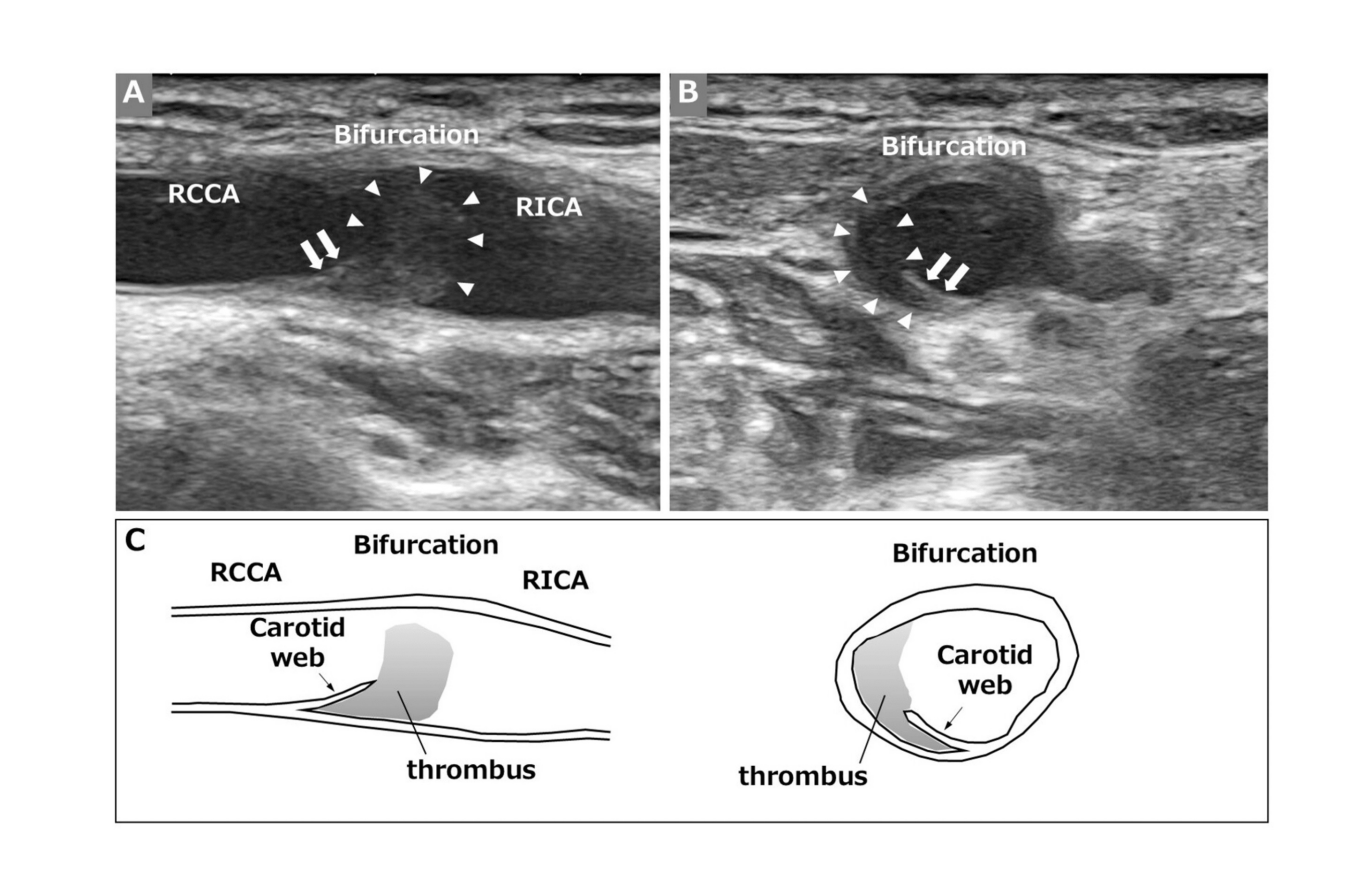
Postoperative carotid ultrasonography. Carotid ultrasonography (A: longitudinal view, B: axial view) reveals a membrane-like structure projecting into the lumen from the posterior wall of the right carotid bifurcation (arrows) with a lodged low-echoic thrombus (arrowheads). An illustration (C) provides a schematic representation of the carotid ultrasonography findings. The illustration was created by the authors. RCCA, right common carotid artery; RICA, right internal carotid artery

However, postoperative carotid ultrasonography identified a membrane-like structure with a lodged thrombus at the posterior wall of the RCCA (Figures 3A-3C), leading to a diagnosis of cerebral embolism due to symptomatic CaW. The thrombus contained some mobile components (not shown in the figure).

**Figure 4:**
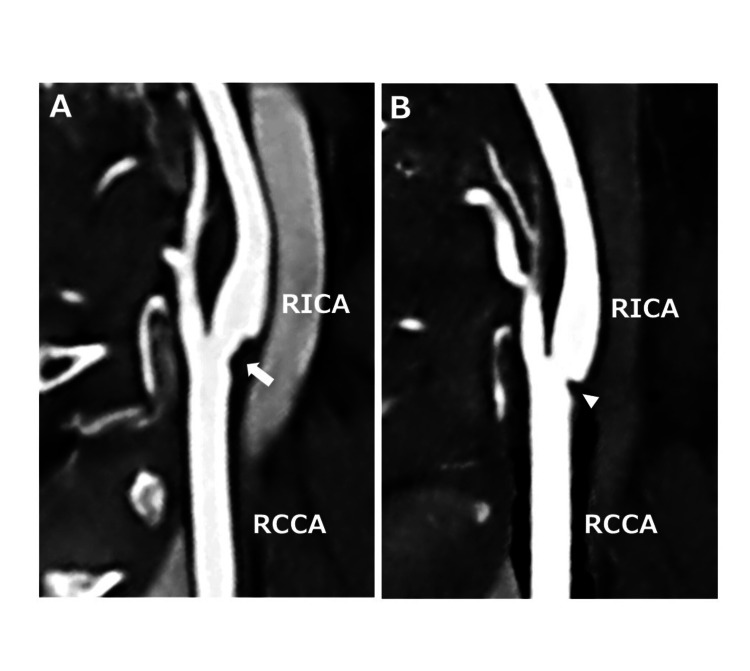
Three-dimensional CT angiography was performed after thrombectomy and one year before this admission. Sagittal multi-planar reconstruction of three-dimensional CT angiography performed after thrombectomy (A) reveals mild, insignificant (<50%) stenosis at the posterior wall of the right carotid bulb (arrow). Whereas, a shelf-like filling defect, suggestive of a carotid web, is recognized in three-dimensional CT angiography one year prior to this admission (B) (arrowhead). RCCA, right common carotid artery; RICA, right internal carotid artery

3D-CT angiography revealed only mild, insignificant (<50%) stenosis at the RCCA bifurcation and failed to identify the CaW (Figure 4A). Upon reviewing the previous 3D-CT angiography from the hospital where she had been treated for subarachnoid hemorrhage a year earlier, we confirmed the presence of a shelf-like filling defect suggestive of a CaW (Figure 4B).

**Figure 5:**
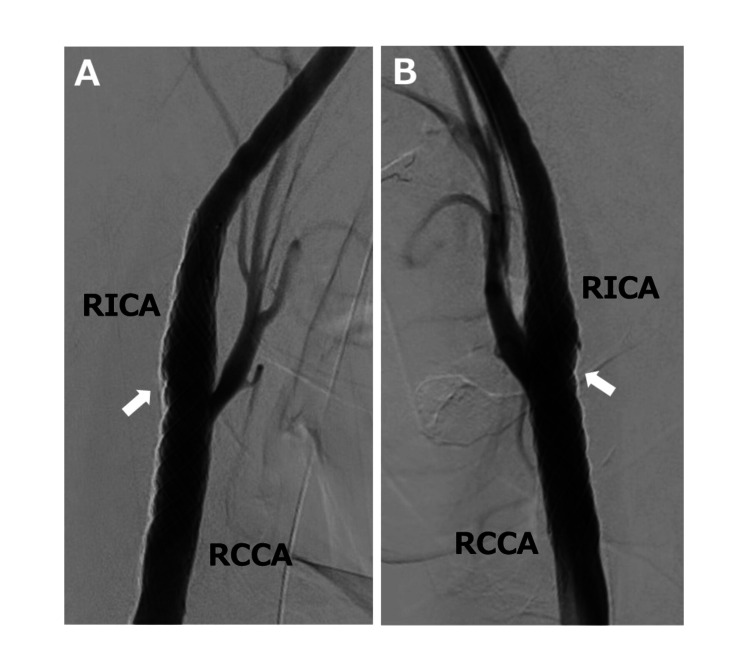
Digital subtraction angiography after carotid artery stenting. A filling defect caused by a carotid web and lodged thrombus at the posterior wall of the right carotid bulb diminished after carotid artery stenting (arrow) (A: anterolateral view, B: lateral view). RCCA, right common carotid artery; RICA, right internal carotid artery.

Intravenous heparin anticoagulation and antiplatelet therapy with clopidogrel (75 mg) and aspirin (100 mg) were initiated. On day five, carotid artery stenting (CAS) was performed to prevent further cerebral embolism (Figures 5A, 5B). The postoperative course was uneventful, and she was transferred to another hospital for rehabilitation on day 33.

## Discussion

To our knowledge, this is the first case report of the symptomatic carotid web that was missed on DSA and 3D-CT angiography due to a lodged thrombus but identified through carotid ultrasonography. Because CaW is considered a variant of fibromuscular dysplasia, and carotid artery fibromuscular dysplasia is sometimes associated with the development of intracranial saccular aneurysms [4], the previous subarachnoid hemorrhage due to a ruptured right internal carotid-posterior communicating artery aneurysm may be related to the symptomatic CaW in this case.

Diagnosing CaW can be challenging because the thin membranous structure typically does not cause hemodynamically significant stenosis and is often missed by routine neuroimaging. It may mimic arterial dissection, non-calcified atherosclerotic plaque, or intraluminal thrombus, leading to potential misdiagnosis [3]. DSA and 3D-CT angiography, which can detect shelf-like filling defects, are considered the gold standard for diagnosing CaW, whereas carotid ultrasonography is generally reported to be less accurate [1-3,5-7]. However, when a large thrombus is lodged beneath the CaW, as in this case, DSA or 3D-CT angiography may show only a slight, insignificant (<50%) filling defect, potentially leading to a missed diagnosis. Additionally, a lodged thrombus may conceal contrast retention in the venous phase, a characteristic finding of CaW on DSA [5]. Because thrombus formation with CaW is not uncommon, occurring in approximately 16-29% of cases [8,9], clinicians should be aware that a lodged thrombus can obscure the diagnosis of CaW using contrast-based angiography. Carotid ultrasonography can be particularly useful in such scenarios, highlighting the importance of multimodal imaging for detecting CaW in patients with embolic strokes of undetermined source (ESUS). Since some CaW cases, like the present one, involve large thrombi and carry a high risk of recurrent ischemic stroke, early screening for CaW is recommended in ESUS, particularly in younger patients who present with an insignificant (< 50%) filling defect at the carotid bifurcation on contrast-angiography and lack vascular risk factors.

The optimal treatment for symptomatic CaW has not yet been established. Medical treatment alone, whether anticoagulation or antiplatelet therapy, may not suffice in preventing stroke recurrence, as recurrence rates remain high, occurring in approximately 16-29% of cases [9,10]. Since both carotid endarterectomy (CEA) and CAS have been reported to be effective and safe for treating symptomatic CaW [7-11], these revascularization procedures can be recommended to prevent ischemic stroke recurrence [7-11]. Therefore, in cases of ESUS, it is crucial to avoid missed diagnoses of symptomatic CaW through multimodal imaging and to pursue revascularization with CEA or CAS to prevent ischemic stroke recurrence.

## Conclusions

We presented a case of symptomatic CaW that was missed using DSA and 3D-CT angiography due to a lodged thrombus but successfully identified through carotid ultrasonography. CaW is an important embolic source in ESUS cases, and DSA or CTA is recognized as the gold standard method for its diagnosis. However, this case has demonstrated that when CaW is associated with a lodged thrombus, it may be missed on DSA or CTA, making the diagnosis challenging. Clinicians should note that a lodged thrombus can be a pitfall in diagnosing CaW using contrast-based angiography techniques. Carotid ultrasonography can be particularly useful in such cases, and screening for CaW with multimodal imaging, including ultrasonography, is essential in younger patients with ESUS, particularly those without vascular risk factors. Since revascularization procedures with CEA or CAS are highly effective in preventing the recurrence of ischemic stroke in symptomatic CaW, it is crucial to avoid missed diagnoses of symptomatic CaW in cases of ESUS through multimodal imaging and to pursue revascularization with CEA or CAS to prevent ischemic stroke recurrence.
